# A Multicenter Study Evaluating the Stages of Change in Food Consumption with Warning Labels among Chilean University Students

**DOI:** 10.1155/2020/2317929

**Published:** 2020-01-16

**Authors:** Samuel Durán Agüero, Jacqueline Araneda, Danay Ahumada, Jaime Silva Rojas, Rodrigo Bühring Bonacich, Astrid Caichac, Marcelo Fernández Salamanca, Pía Villarroel, Eloína Fernandez, Viviana Pacheco, Paola Aravena Martinovic, Waleska Wilson, Ana María Neira, Claudia Encina, Jessica Moya Tillería

**Affiliations:** ^1^Escuela de Nutrición y Dietética, Facultad de Ciencias para el Cuidado de la Salud, Universidad San Sebastián, Chile; ^2^Departamento de Nutrición y Salud Pública, Facultad de Ciencias de la Salud y de los Alimentos, Universidad del Bío-Bío, Chillán, Chile; ^3^Departamento de Procesos Diagnósticos y Evaluación, Facultad de Ciencias de la Salud, Universidad Católica de Temuco, Chile; ^4^Facultad de Ciencias de la Salud, Universidad de Tarapacá, Chile; ^5^Departamento de Salud Pública, Facultad de Medicina, Universidad Católica de la Santísima Concepción, Chile; ^6^Carrera de Nutrición y Dietética, Universidad Autónoma de Chile, Chile; ^7^Escuela de Nutrición y Dietética, Facultad de Medicina, Universidad Andres Bello, Chile; ^8^Escuela de Nutrición y Dietética, Facultad de Ciencias, Universidad Mayor, Chile; ^9^Departamento Cs. De los Alimentos y Nutrición, Facultad Ciencias de la Salud, Universidad de Antofagasta, Chile; ^10^Carrera de Nutrición y Dietética, Facultad de Ciencias de la Salud, Universidad de Magallanes, Chile; ^11^Escuela de Nutrición y Dietética, Facultad de Ciencias de la Salud, Universidad de Talca, Chile; ^12^Escuela de Nutrición y Dietética, Universidad Bernardo O'Higgins, Chile; ^13^Escuela de Nutrición y Dietética, Facultad de Salud, Universidad Santo Tomás, sede Viña del Mar, Chile

## Abstract

**Objective:**

To analyze the stage of change in food consumption with warning labels among Chilean university students.

**Materials and Methods:**

Cross-sectional study which applied surveys in universities from all over the country. Study included 4807 participants of 18 to 40 years of both sexes who were asked about the level of knowledge of the new food law and food consumption with warning signals, including questions regarding their willingness to behavior change according to Prochaska's transtheoretical model. To compare continuous variables, Student's t-test was used in the statistical package SPSS 22.0, and p <0.05 was considered a significant difference.

**Results:**

Of the total number of respondents, 99.3% of the students indicated that they know about the food law, classifying foods with signals in the precontemplation stage. Compared by sex, we observed that women give greater importance to behavior change in all of foods (p <0.001). Underweight students give less importance to change in unhealthy foods, while obesity students give more importance but do not show more confidence in behavior change (p <0.05).

**Conclusion:**

The university students show a low importance and confidence to make behavior change, aspects associated with sex and nutritional status. It is necessary to strengthen nutritional food education and not just talk about structural measures.

## 1. Introduction

Worldwide changes in dietary patterns towards greater consumption of highly processed foods have been well recognized [1, 2].

National data from Chile shows the consequences of these bad eating habits. According to the 3rd National Health Survey, people between 20 and 24 years of age have a 33.6% prevalence of low HDL, 20.8% high triglycerides, 9.6% high total cholesterol, 32.9% elevated LDL cholesterol, 13.6% metabolic syndrome (age range 15-24), 18.4% moderate to high cardiovascular risk (age range 15-24), 2.1% morbid obesity, 22.5% obesity, and 35.8% overweight (age range 20-29) [3].

College students, who are found in this age group, are particularly vulnerable to unhealthy eating environments, have poor eating habits, often eat between meals, fast for many hours during the day, do not eat breakfast, and prefer fast food, rich in saturated fat, sugar, and salt [4, 5].

Food preferences relate to the fact that in this period college students are responsible for selecting their meals, the patterns, and frequency of consumption of each food [6]. Eating habits are characterized by a low consumption of fruits, which is related to long hours of study, classes at diverse times, increases in nightlife, and a scarce budget, among other factors. Low fruit consumption can consequently worsen overall diet and nutritional status [7–11].

In addition to the above, Chile is the largest consumer of sugar-sweetened beverages in the world, displacing Mexico and the United States [12] and one of the largest consumers of ultra-processed foods [13], foods that are particularly desired by young people.

Considering the current food problem in Chile, on June 26, 2016, Law 20.606, the Chilean Law of Food Labeling and Advertising came into force. The law has 3 main parts: (i) bans on the sale of foods in schools, (ii) advertising bans aimed at children under 14 years of age, and (iii) warning labeling on foods; the latter is indicated in decree 13/2015 of the Ministry of Health [14]. The main characteristic of this law is the use of warning signals on the front of packaging represented by 4 black octagons with white letters when levels of critical nutrients (calories, saturated fats, sugars, and sodium) exceed the allowed limits. A particular food may have up to 4 warning labels if levels of each critical nutrients exceed recommendations.

The Transtheoretical or Stages of Change Model in health behavior was first described in 1979 by James Prochaska and was consolidated during the 1990s as one of the most innovative proposals in the area of health promotion and disease prevention [15]. It offers possibilities to plan and execute interventions based on the stage of change the populations, for whom actions are directed, finding themselves in precontemplation, contemplation, preparation, action, and maintenance. Several publications recognize the ability of the model to describe and explain the different stages that are common to most processes of behavioral change and that, at the beginning of the 1990s, were progressively incorporated into the investigations and interventions of a large number of behaviors already recognized as health risks [15]. The American College of Sports Medicine adapted the classic model of evaluation of Prochaska's stages of change to include the importance placed on making a change and the confidence to do so. Precontemplation: the person did not express interest in adopting a certain habit. Contemplation: the person expressed interest to change their behavior and adopt the habit but was not willing to do so in the next 6 months. Preparation for action: the person was willing to adopt a habit within the next 30 days and had also incorporated certain habits or isolated actions leading to behavior change. Action: the person adopted a behavior less than 6 months ago. Maintenance: the person had maintained the behavior for more than 6 months.

The objective of the present study was to analyze the stage of behavioral change among university students to stop eating foods that contain warning labels and compare results by sex and nutritional status.

## 2. Material and Methods

We conducted a cross-sectional study. Surveys were created in Google Forms and sent to university students from all over the country. Chilean men and women between 18 and 40 years old were invited to participate. Exclusion criteria: persons with a visual disability. The population of university students in Chile was used for the sample size calculation. With a 95% confidence level and 3% error margin, we calculated that a sample of 1067 students was needed. The study was developed following the Declaration of Helsinki regarding working with human participants and approved by the Ethics Committee at San Sebastián University.

**Figure 1:**
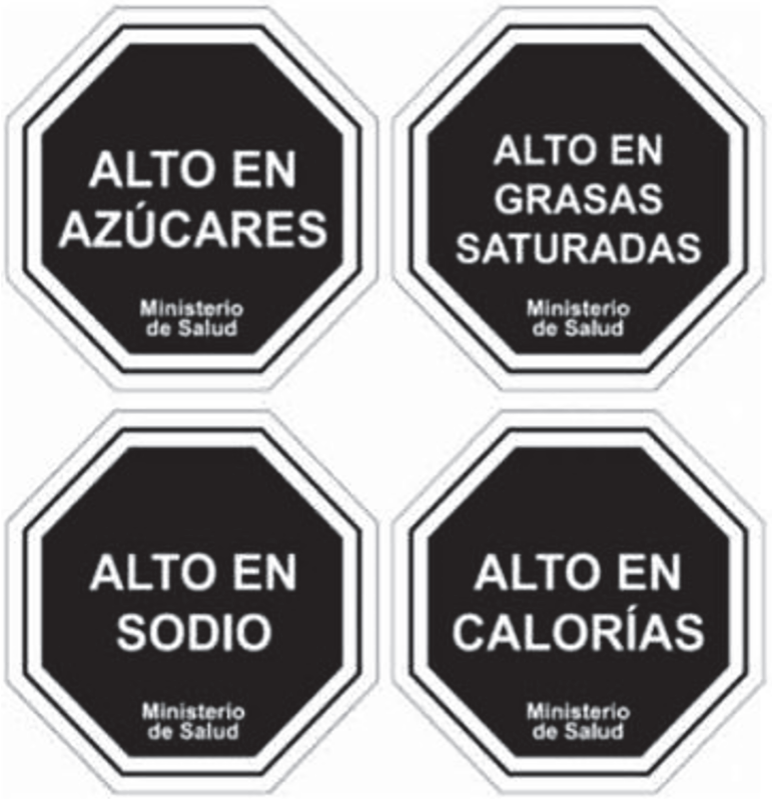
Warning labels for foods “HIGH IN” critical nutrients (clockwise starting from upper left: sugar, saturated fats, calories, and sodium).

Participants provided informed consent prior to the start of the study, after which sex, age, weight, height, college major, and university were asked. Next, participants were asked if they were aware of the new food labeling law (Law 20.606), if they were aware that the law allows for the identification of foods with critical nutrients, and if they understood what that exceed critical nutrients (Figure 1).

In addition, the consumption of foods containing warning labels was evaluated. Prior to the beginning of the study, 10 foods that had 1 or more warning labels were selected via expert opinion. The selected foods were carbonated drinks, sugary juices, cookies, sweet snacks, French fries, chocolate, cold cuts, soups, ice cream, and breakfast cereals.

If a student reported eating a food with a warning label, they answered two additional questions related to desire to make a change in behavior: (1) how important is it for you to stop consuming the food? (scale of 1 to 10, with 1 meaning “not important” and 10 “very important”); (2) how confident do you feel that you will be able to stop consuming the food (scale of 1 to 10, with 1 meaning “not confident” and 10 “very confident”).

The questions corresponded to the adaptation made by the American College of Sports Medicine to the classic model of evaluation of the stages of change. Recently, the scales to measure importance and confidence have been recognized by the American Academy of Nutrition and Dietetics as very effective to achieve changes in eating behavior [16]. Both assigned scores are crossed in a grid and determine the stage of change of the subject.

### 2.1. Statistical Analysis

For continuous variables, the Kolmogorov-Smirnov normality test was used. For comparisons between groups Student's t-test was used. For comparisons between groups, analysis of variance and Bonferroni post hoc tests were performed. The statistical package SPSS 22.0 was used and p <0.05 was considered statistically significant.

## 3. Results

We interviewed 4807 university students from all over the country, 23.8% were male, with an average age of 22.2±3.5 years, weight of 65.3±13.0 kg, and BMI of 24.0±3.8 kg/m^2^.  Table 1 describes the nutritional characterization of the sample.

**Figure 2:**
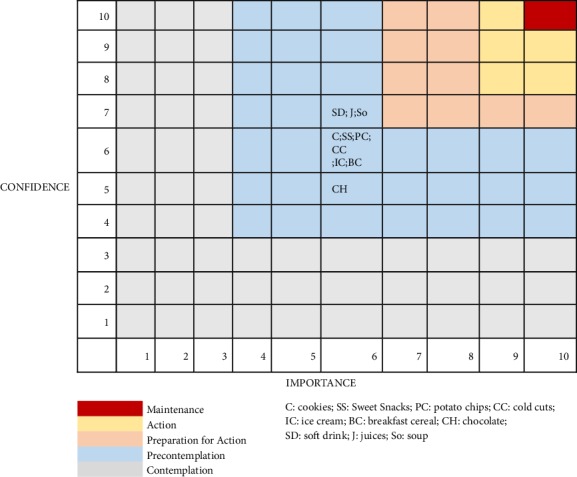
Stage of change based on confidence and importance of changing consumption of particular foods.

Nearly all students (99.3%) reported knowing about the food labeling law, 97.9% indicated that the law allows for the identification of foods with excess critical nutrients, and 96.4% indicated knowing when foods contained high content of calories, sodium, saturated fats, and sugar. Average scores for participant regarding the importance of stopping consumption of particular foods and how confident they were that they could make this change are described in Figure 2. We observed that students were classified in the precontemplation stage.

When comparing by sex we observed that women, compared to men, reported higher levels of importance to change consumption of all the foods evaluated (p <0.001). However, with respect to confidence in their ability to make reduce consumption, they had higher scores, compared to men, for juices, cold cuts, soups, and breakfast cereals (p <0.001) (Table 2).

When comparing the importance to stop eating particular foods and confidence to do so by nutritional status, we observed that students with low weight reported lower importance to change consumption of certain products, such as chocolates and sweet snacks, and lower confidence in their ability to stop consuming cookies that had warning labels. Students with obesity were less confident in their ability to stop consuming sugary drinks (p<0.05) (Table 3).

When comparing nutritional status within the group of women (Table 4), we observed that women with low weight gave less importance to changing consumption of cookies, sweets, chips, and chocolates. The opposite occurred with obese women. Obese women had lower confidence in their ability to stop eating: ice cream, cold cuts, and soft drinks.

On the other hand, when comparing according to the nutritional status among men (Table 5), we observed that men with low weight gave less importance to stopping consumption of soft drinks, sweets, and chocolates and had less confidence in their ability to stop consuming cookies, sweets, and soft drinks. Obese men placed the greatest importance on changing habits related to soft drink consumption.

## 4. Discussion

The main result of this study was that participants placed a low importance on the need to stop eating foods with warning labels and had low confidence that they could do so. Underweight university students gave low importance to making changes in the purchase of unhealthy foods, and students with obesity reported that making changes was more important, but they were not confident in their ability to change behavior.

Women, compared to men, placed higher importance on stopping the consumption of foods with front-of-the-package warning labels and in some foods women had greater confidence, compared to men, that they could make changes. Several studies show that women have healthier patterns than men [8–11].

It is interesting to note that underweight students of both sexes had the lowest scores in both the importance and the confidence to stop consuming ultra-processed foods, probably because they feel that it is not necessary to make changes to an unhealthy diet because they need to consume more calories. On the other hand, students with obesity had the highest scores in importance to stop eating these products, which were different according to sex, with women concerned about reducing the consumption of cookies, sweets, chips, and chocolate and men of soft drinks only.

The consumption of ultra-processed foods in Chile has increased in recent decades, and Chile is among the largest consumers of these products, especially sugary beverages. These foods represent 28.6% of total energy intake and 58.6% of intake of added sugars [16]. The high consumption of these foods has been associated with cancer [17], obesity [18, 19], and cardiovascular disease [20, 21]. In addition, these foods are very present in the media and use large marketing campaigns in all media platforms. Children and youth are the most vulnerable populations for the marketing of these products [22].

In Chile, 86.9% of people require changes in their eating habits and 7.8% eat an unhealthy diet [23, 24]. As a way to stop this problem, Chile created the food labeling law. One of the main parts of the law is the use of front-of-package warning labels represented by black octagons with white letters that alert the consumer if critical nutrients (calories, saturated fats, sugars, and sodium) exceed the permitted limits. These “HIGH IN” warning labels allow the consumer to distinguish with a glance which foods are less healthy, in order to be able to choose foods without warning labels or choose to eat less of the foods with warning labels.

Some studies have shown a decrease in the consumption of certain foods since the law has been in effect. One study was conducted after the labeling law had been in effect for 6 months and showed that consumer purchase preferences did seem to have changed and that consumers considered the number of warning labels when buying a product [25]. Brazil has also implemented a regulation act for school cafeterias; this law has been evaluated several times. One of the evaluations took place 7 years after the implementation of the law in the State of Santa Catarina, which has 8 townships. When the study was carried out, in more than half of these townships school cafeterias continued to sell food with a poor nutritional content and prohibited by the law, like pizzas, chocolates, and chocolate drinks [26]. Another country which has also implemented food regulations is Uruguay, implementing in 2013 the law 19.140 “Alimentación saludable en los centros de enseñanza” (“Healthy diet at educational institutions”). The aim of this law is to protect the health of children and adolescents who attend educational establishments through the promotion of healthy eating habits in the educational context, as a mechanism to contribute to prevention of chronic diseases, acting against this risk factor [27]. In 2015, researchers carried out an evaluation of the law enforcement, assessing the availability of healthy products in supermarkets; inappropriate products offering was still higher than appropriate products aimed for the consumption as school snacks. This limitation is a problem when it comes to choose recommended food products, whether these products are going to be sold at school cafeterias or consumed by the families [27].

Despite these results, there is a large population that resists change, as was demonstrated in the current study. Participants in our study knew the law and identified the purpose of the warning labels. Another study reported that 81% of those sampled indicated that the law required improvements, for example, the need for more education, in terms of the health impacts of consumption of certain foods [26]. This high desire for a change in the law may be reflective of a precontemplative stage for change in consumption habits which was demonstrated in our study.

It should be noted that, at the end of June 2018, “Phase 2” of the implementation of Law 20.606 will begin. This new phase corresponds to a second stage in the application of this regulation, in which the maximum limits will be reduced, both for solid and liquid foods. Thus, foods that before June 2018 were free of warning labels and may begin to have labels after the implementation of the second phase of the law, which may favor changes in behavior in the general population.

Among the strengths of this study is that we worked with a previously validated model and used a national sample of university students. Among the weaknesses, we acknowledge that because this was a cross-sectional study we can only discuss our findings in terms of associations and not causal relationships.

## 5. Conclusions

In this study, university students showed both a low importance and confidence to make changes in their food habits, specifically to stop consumption of foods with warning labels. In addition, sex and nutritional status were associated with stage of change for importance and confidence. Underweight students gave little importance to making changes in the consumption of unhealthy foods, while students with obesity gave more importance, but did not have more confidence in behavior change. It is necessary to strengthen nutritional food education starting in early life and not just talk about structural measures.

## Figures and Tables

**Table 1:** Nutritional characteristics of the sample (mean ± SD).

	Men	Women	P-value
	(n=1,365)	(n=3,442)	
Age (years)	22.5 ± 3,9	22.1 ± 3.3	0.001
Weight (kg)	80.3 ± 13.7	66.0 ± 11.6	0.001
Height (m)	1.72 ± 0.07	1.59 ± 0.09	0.001
BMI (kg/m^2^)	26.9 ± 4.2	26.1 ± 4.7	0.001

**Table 2:** Comparisons of the importance and confidence to change consumption of particular foods by sex.

	Men	Women	P-value
	(n=1365)	(n=3442)	
Soft drinks			
Importance	6.5 ± 3.2	6.9 ± 3.3	0.001
Confidence	7.1 ± 2.8	7.6 ± 2.7	0.001
Juices			
Importance	5.9 ± 3.2	6.6 ± 3.2	0.001
Confidence	6.6 ± 3.0	7.3 ± 2.7	0.001
Cookies			
Importance	6.1 ± 2.8	6.7 ± 2.9	0.001
Confidence	6.0 ± 3.2	6.7 ± 3.0	0.962
Sweet snacks			
Importance	6.3 ± 3.3	7.0 ± 3.1	0.001
Confidence	6.5 ± 3.0	6.5 ± 2.9	0.895
Potato chips			
Importance	6.7 ± 3.1	6.7 ± 3.0	0.001
Confidence	5.8 ± 2.9	6.2 ± 2.8	0.847
Cold cuts			
Importance	5.8 ± 3.2	6.9 ± 3.2	0.001
Confidence	5.8 ± 3.1	7.1 ± 2.9	0.001
Soups			
Importance	6.2 ± 3.6	6.8 ± 3.6	0.001
Confidence	7.5 ± 3.1	8.0 ± 2.9	0.001
Chocolate			
Importance	5.9 ± 3.3	6.6 ± 3.3	0.001
Confidence	5.7 ± 3.5	6.5 ± 3.2	0.171
Ice-cream			
Importance	5.7 ± 3.3	6.5 ± 3.2	0.001
Confidence	6.1 ± 3.2	6.3 ± 3.1	0.082
Breakfast cereals			
Importance	5.7 ± 3.5	6.6 ± 3.4	0.001
Confidence	6.4 ± 3.3	7.0 ± 3.1	0.001

Values expressed as mean ± SD. Student t-test.

**Table 3:** Comparisons of the importance and confidence to change consumption of particular foods by nutritional status.

	Under weight	Normal weight	Overweight	Obese
	(n=158)	(n=3007)	(n=1195)	(n=340)
Soft drinks				
Importance	6.4 ± 3.3	6.7 ± 3.3	6.8 ± 3.2	6.9 ± 3.1
Confidence	7.1 ± 2.9	7.5 ±2.7	7.4 ± 2.7	7.0 ± 2.7^a^
Juices				
Importance	6.1 ± 3.2	6.4 ± 3.2	6.5 ± 3.1	6.3 ± 3.1
Confidence	6.9 ± 2.9	7.1 ± 2.8	7.3 ± 2.8	6.7 ± 2.8
Cookies				
Importance	5.5 ± 3.2	6.4 ± 3.1	6.6± 3.0	6.9 ± 2.8
Confidence	5.6 ± 3.2^a^	6.2 ± 2.9	6.3 ± 2.8	6.0 ± 2.8
Sweet snacks				
Importance	6.0 ± 3.3^a^	6.7 ± 3.2	7.0 ± 3.0	7.2 ± 2.9
Confidence	6.0 ± 3.2	6.5 ± 3.0	6.6 ± 2.9	6.3 ± 3.5
Potato chips				
Importance	6.3 ± 3.5	6.8 ± 3.3	7.0 ± 3.1	7.3 ± 3.0
Confidence	6.0 ± 3.4	6.7 ± 3.0	6.8 ± 3.0	6.7 ± 3.0
Cold cuts				
Importance	6.1 ± 3.4	6.6 ± 3.3	6.5 ± 3.2	6.6 ± 3.0
Confidence	6.6 ± 3.3	6.8 ± 3.0	6.6 ± 3.0	6.1 ± 3.0
Soups				
Importance	6.4 ± 3.6	6.7 ± 3.6	6.6 ± 3.6	6.6 ± 3.6
Confidence	7.5 ± 3.2	7.9 ± 2.9	7.8 ± 3,0	7.8 ± 3.0
Chocolate				
Importance	5.7 ± 3.6^a^	6.2 ± 3.3	6.7 ± 3.2	6.9 ± 3.1
Confidence	5.4 ± 3.5	5.5 ± 3.3	5.7 ± 3.2	5.4 ± 3.2
Ice-cream				
Importance	5.7 ± 3.4	6.2 ± 3.3	6.5 ± 3.2	6.6 ± 3.2
Confidence	5.5 ± 3.4	6.3 ± 3.1	6.3 ± 3.1	6.2 ± 3.1
Breakfast cereals				
Importance	6.0 ± 3.5	6.3 ± 3.4	6.4 ± 3.5	6.3 ± 3.4
Confidence	5.9 ± 3.3	6.8 ± 3.2	7.0 ± 3.2	6.7 ± 3.2

Values expressed as mean ± SD. Anova test post hoc Bonferroni. Equal letters indicate significant differences.

**Table 4:** Comparisons of the importance and confidence to change consumption of particular foods by nutritional status among women.

	Under weight	Normal weight	Overweight	Obese
	(n=135)	(n=2225)	(n=781)	(n=234)
Soft drinks				
Importance	6.5 ± 3.3	6.9 ± 3.3	6.9 ± 3.5	6.9 ± 3.2
Confidence	7.2 ± 2.8	7.7 ± 2.6^a^	7.4 ± 2.7	7.1 ± 2.7^a^
Juices				
Importance	6.1 ± 3.2	6.6 ± 3.2	6.7 ± 3.1	6.5 ± 3.2
Confidence	7.0 ± 2.8	7.3 ± 2.7	7.4 ± 2.7	6.9 ± 2.8
Cookies				
Importance	5.6 ± 3.2^a^	6.6 ± 3.0	6.8 ± 2.9	7.1 ± 2.7^a^
Confidence	5.7 ± 3.2	6.2 ± 2.9	6.2 ± 2.8	6.0 ± 2.8
Sweet snacks				
Importance	6.1 ± 3.3^a^	6.9 ± 3.1	7.3 ± 2.9	7.4 ± 2.7^a^
Confidence	6.0 ± 3.1	6.5 ± 3.0	6.5 ± 2.8	6.2 ± 2.9
Potato chips				
Importance	6.3 ± 3.4^a^	7.0 ± 3.2	7.1 ± 3.1	7.4 ± 2.9^a^
Confidence	5.9 ± 3.3^a,b,c^	6.8 ± 3.0^b^	6.7± 2.9^c^	6.8 ± 2.9^a^
Cold cuts				
Importance	6.2 ± 3.4	6.9 ± 3.2	6.8 ± 3.2	6.9 ± 3.0
Confidence	6.8 ± 3.1	7.2 ± 2.9^a^	7.0 ± 2.9	6.5 ± 3.0^a^
Soups				
Importance	6.5 ± 3.5	6.9 ± 3.6	6.8 ± 3.6	6.5 ± 3.6
Confidence	7.6 ± 3.1	8.0 ± 2.8	7.8 ± 2.9	7.8 ± 3.0
Chocolate				
Importance	5.6 ± 3.5^a^	6.4 ± 3.3	6.9 ± 3.1	7.0 ± 3.1^a^
Confidence	5.3 ± 3.4	5.5 ± 3.3	5.7 ± 3.2	5.2 ± 3.2
Ice-cream				
Importance	5.7 ± 3.4	6.3 ± 3.1	6.7 ± 3.2	6.8 ± 3.1
Confidence	5.4 ± 3.3^a^	6.3 ± 3.1^a^	6.4 ± 3.0^a^	6.4 ± 3.0^a^
Breakfast cereals				
Importance	6.1 ± 3.4	6.6 ± 3.4	6.7 ± 3.4	6.5 ± 3.4
Confidence	5.9 ± 3.3^a^	6.8 ± 3.2	7.3 ± 3.0^a^	6.8 ± 3.1

Values expressed as mean ± SD. Anova test post hoc Bonferroni. Equal letters indicate significant differences.

**Table 5:** Comparisons of the importance and confidence to change consumption of particular foods by nutritional status among men.

	Under weight	Normal weight	Overweight	Obese
	(n=23)	(n=782)	(n=414)	(n=106)
Soft drinks				
Importance	5.8 ± 3.6^a^	6.2 ± 3.3	6.8 ± 3.1	7.0 ± 2.9^a^
Confidence	6.6 ± 3.4^a^	6.9 ± 2.9	7.4 ± 2.7^a^	6.7 ± 2.8
Juices				
Importance	6.2 ± 3.6	5.7 ± 3.2	6.1 ± 3.1	5.8 ± 3.0
Confidence	6.3 ± 3.6	6.5 ± 3.0	7.0 ± 2.9	6.3 ± 2.9
Cookies				
Importance	5.2 ± 3.3	5.9 ± 3.2	6.2± 3.2	6.6 ± 3.0
Confidence	4.9 ± 3.3^a^	6.0 ± 3.0	6.5 ± 2.8^a^	6.0 ± 3.0
Sweet snacks				
Importance	5.7 ± 3.8^a^	6.0 ± 3.4	6.6 ± 3.2	6.7 ± 3.3^a^
Confidence	6.3 ± 3.0	6.4 ± 3.7	6.7 ± 2.9	6.5 ± 3.2
Potato chips				
Importance	6.1 ± 3.7	6.3 ± 3.4	6.8 ± 3.2	7.0± 3.3
Confidence	6.4 ± 3.6	6.6 ± 3.1	7.0 ± 3.0	6.7± 3.2
Cold cuts				
Importance	5.4 ± 3.8	5.7 ± 3.2	6.0 ± 3.2	5.9 ± 3.0
Confidence	5.5 ± 3.8	5.7 ± 3.1	6.0 ± 3.0	5.3 ± 3.0
Soups				
Importance	5.7 ± 3.8	6.2 ± 3.7	6.1 ± 3.7	6.9 ± 3.4
Confidence	7.3 ± 3.6	7.4 ± 3.2	7.6 ± 3.1	7.7 ± 3.0
Chocolate				
Importance	5.8 ± 3.9^a^	6,4 ± 3,3	6.2 ± 3.3	6.5 ± 3.3^a^
Confidence	5.5 ± 3.3	5.9 ± 3.8	5.9± 3.2	5.8 ± 3.3
Ice-cream				
Importance	5.4 ± 3.3	5.8 ± 3.7	5.9± 3.3	5.8 ± 3.3
Confidence	6.1 ± 3.2	5.7 ± 3.7	6.3 ± 3.2	5.9 ± 3.3
Breakfast cereals				
Importance	5.6 ± 4.0	5.6 ± 3.5	5.8 ± 3.5	6.0 ± 3.3
Confidence	5.7 ± 3.7	6.3 ± 3.3	6.5 ± 3.3	6.6 ± 3.3

Values expressed as mean ± SD. Anova test post hoc Bonferroni. Equal letters indicate significant differences.

## Data Availability

The data used to support the findings of this study are available from the corresponding author upon request.

## Supplementary Materials

Supplementary Materials Table 6 shows that most students are in the precontemplation stage for most foods. When comparing stages of change regarding the purchase of packaged foods we can observe that obese students are mostly in the precontemplation stage in the item sugar-sweetened beverages. In contrast, in the categories of sweet snacks, potato chips, chocolate and cured meats, and sausages, underweight students are the ones with the greater proportion in the precontemplation stage. Figure 3 shows that, irrespective of gender, most subjects are in the precontemplation stage in all food categories. Men are in precontemplation mostly in the categories of breakfast cereals, ice cream, instant soups, sweet snacks, cookies, and juice with sugar, while women are mostly in precontemplation in sugar-sweetened beverages.
