# Supplementary material for: A Multicenter Study Evaluating the Stages of Change in Food Consumption with Warning Labels among Chilean University Students
Source: Biomed Res Int. 2020 Jan 16;2020:2317929. doi: 10.1155/2020/2317929 (PMC7212336; doi:10.1155/2020/2317929)
Supplement: Supplementary Materials — Table 6 shows that most students are in the precontemplation stage for most foods. When comparing stages of change regarding the purchase of packaged foods we can observe that obese students are mostly in the precontemplation stage in the item sugar-sweetened beverages. In contrast, in the categories of sweet snacks, potato chips, chocolate and cured meats, and sausages, underweight students are the ones with the greater proportion in the precontemplation stage. Figure 3 shows that, irrespective of gender, most subjects are in the precontemplation stage in all food categories. Men are in precontemplation mostly in the categories of breakfast cereals, ice cream, instant soups, sweet snacks, cookies, and juice with sugar, while women are mostly in precontemplation in sugar-sweetened beverages. [file 2317929.f1.docx]

Table 6: Comparison of the stages of change for the purchase of packaged food with warning labels by nutritional status

|  |  | Precontemplation | Contemplation | Preparation for action | Action |
| --- | --- | --- | --- | --- | --- |
| Sugar-sweetened beverages | Normal | 821 (37.0) | 667 (30.1) | 361 (16.3) | 369 (16.6) |
| Chi-Square Test, p value=**0.004** | Under weight | 46 (38.7) | 41 (34.5) | 19 (16.0) | 13 (10.9) |
|  | Overweight | 327 (35.9) | 254 (27.9) | 173 (19.0) | 158 (17.3) |
|  | Obese | 83 (30.4) | 101 (37.0) | 55 (20.1) | 34 (12.5) |
| Juice with sugar | Normal | 824 (37.1) | 822 (37.0) | 362 (16.3) | 214 (9.6) |
| Chi-Square Test, p value=0.244 | Under weight | 48 (40.0) | 45 (37.5) | 14 (11.7) | 13 (10.8) |
|  | Overweight | 304 (34.3) | 332 (37.5) | 156 (17.6) | 94 (10.6) |
|  | Obese | 93 (34.1) | 113 (41.4) | 42 (15.4) | 25 (9.2) |
| Cookies | Normal | 824 (37.1) | 822 (37.0) | 362 (16.3) | 214 (9.6) |
| Chi-Square Test, p value=0.226 | Under weight | 48 (39.7) | 45 (37.2) | 14 (11.6) | 14 (11.6) |
|  | Overweight | 304 (34.3) | 332 (37.5) | 156 (17.6) | 94 (10.6) |
|  | Obese | 93 (34.1) | 113 (41.4) | 42 (15.4) | 25 (9.2) |
| Sweet snacks | Normal | 917 (38.8) | 826 (35.0) | 420 (17.8) | 199 (8.4) |
| Chi-Square Test, p value=**0.004** | Under weight | 63 (48.8) | 40 (31.0) | 12 (9.3) | 14 (10.4) |
|  | Overweight | 312 (33.1) | 364 (38.6) | 189 (20.0) | 78 (8.3) |
|  | Obese | 100 (36.1) | 98 (35.4) | 47 (17.0) | 32 (11.6) |
| Potato Chips | Normal | 898 (39.3) | 753 (33.0) | 378 (16.6) | 254 (11.1) |
| Chi-Square Test, p value=**0.001** | Under weight | 69 (55.2) | 25 (20.0) | 20 (16.0) | 11 (8.8) |
|  | Overweight | 318 (35.1) | 338 (37.3) | 173 (19.2) | 78 (8.6) |
|  | Obese | 86 (34.4) | 92 (36.8) | 48 (19.2) | 24 (9.6) |
| Cured meats and sausages | Normal | 874 (40.7) | 753 (35.1) | 330 (15.4) | 189 (8.8) |
| Chi-Square Test, p value=**0.001** | Under weight | 57 (50.4) | 32 (28.3) | 14 (12.4) | 10 (8.8) |
|  | Overweight | 351 (38.4) | 333 (36.4) | 152 (16.6) | 79 (8.6) |
|  | Obese | 104 (37.4) | 108 (38.8) | 46 (16.5) | 20 (7.2) |
| Instant soups | Normal | 891 (51.3) | 460 (26.5) | 211 (12.2) | 174 (10.0) |
| Chi-Square Test, p value=0.955 | Under weight | 49 (50.5) | 29 (29.9) | 11 (11.3) | 8 (8.2) |
|  | Overweight | 368 (51.0) | 199 (27.6) | 79 (11.0) | 75 (10.4) |
|  | Obese | 103 (51.2) | 55 (27.4) | 25 (12.4) | 18 (9.0) |
| Chocolate | Normal | 1245 (50.0) | 811 (32.6) | 291 (11.7) | 141 (5.7) |
| Chi-Square Test, p value=**0.025** | Under weight | 73 (56.2) | 30 (23.1) | 19 (14.6) | 8 (6.2) |
|  | Overweight | 435 (44.1) | 348 (35.3) | 153 (15.5) | 51 (5.2) |
|  | Obese | 135 (46.7) | 94 (32.5) | 42 (14.5) | 18 (6.2) |
| Ice cream | Normal | 1034 (43.5) | 842 (35.4) | 325 (13.7) | 177 (7.4) |
| Chi-Square Test, p value=0.385 | Under weight | 67 (51.1) | 38 (29.0) | 18 (13.7) | 8 (6.1) |
|  | Overweight | 394 (36.4) | 351 (36.4) | 144 (14.9) | 75 (7.8) |
|  | Obese | 109 (38.8) | 101 (35.9) | 41 (14.6) | 30 (10.7) |
| Breakfast cereals | Normal | 1015 (48.3) | 657 (31.3) | 258 (12.3) | 170 (8.1) |
| Chi-Square Test, p value=0.075 | Under weight | 59 (48.0) | 42 (34.1) | 16 (13.0) | 6 (4.9) |
|  | Overweight | 375 (44.9) | 296 (35.4) | 83 (9.9) | 82 (9.8) |
|  | Obese | 112 (44.4) | 84 (33.3) | 33 (13.1) | 23 (9.1) |

Figure 3: Comparison of the stages of change for the purchase of food with warning labels by sex

Chi-square test, breakfast cereal p<0.001; Ice-cream p<0.001; Chocolate p=0.339; Instant soups p<0.001; Cured meats p<0.001; potato chips p<0.093; sweet snacks p<0.05; cookies p<0.001; Juice with sugar p<0.001; sugar-sweetened beverages p<0.001
